# Effect of Ti/Ni Coating of Diamond Particles on Microstructure and Properties of High-Entropy Alloy/Diamond Composites

**DOI:** 10.3390/e21020164

**Published:** 2019-02-10

**Authors:** Wei Zhang, Mingyang Zhang, Yingbo Peng, Fangzhou Liu, Yong Liu, Songhao Hu, Yang Hu

**Affiliations:** 1 Powder Metallurgy Research Institute, Central South University, Changsha 410083, China; 2 College of Engineering, Nanjing Agricultural University, Nanjing 210031, China; 3 Henan Huanghe Whirlwind Co., Ltd., Xuchang 461500, China; 4 Yuanmeng Precision Technology (Shenzhen) Institute, Shenzhen 518055, China

**Keywords:** high-entropy alloy, diamond, coating, interface, mechanical properties

## Abstract

In this study, an effective way of applying Ti/Ni deposited coating to the surface of diamond single crystal particles by magnetron sputtering was proposed and novel high-entropy alloy (HEA)/diamond composites were prepared by spark plasma sintering (SPS). The results show that the interfacial bonding state of the coated diamond composite is obviously better than that of the uncoated diamond composite. Corresponding mechanical properties such as hardness, density, transverse fracture strength and friction properties of the coated diamond composite were also found to be better than those of the uncoated diamond composite. The effects of interface structure and defects on the mechanical properties of HEA/diamond composites were investigated. The research directions for further improving the structure and properties of high-entropy alloy/diamond composites were proposed.

## 1. Introduction

Multi-principal high-entropy alloys break through the traditional alloy design mode based on one kind of alloy element. By optimizing the composition design, excellent performance combinations such as high strength, high hardness, high temperature creep resistance, high temperature oxidation resistance and corrosion resistance can be obtained [1,2,3,4,5,6,7,8,9]. Based on the excellent properties of high-entropy alloys (HEAs) and diamond, it is of great scientific value and application significance to design a novel high-entropy metal matrix binder for diamond tools and to develop the corresponding theory and technology of heterogeneous multi-phase interface control [10,11,12].

Under the application conditions, improving the interface state between matrix materials and diamond particles is the key problem to be urgently solved in the research field of diamond tool materials. For diamond tools with an HEA matrix, improving the wettability between the HEA matrix and diamond particles and effectively controlling the reaction products of the HEA/diamond interface are important for improving the interface bonding and overall application performance of diamond tools. Ti, Cr, Mo, V and other strong carbide-forming elements were always selected as coating materials [13,14,15], which can form a carbide layer on the surface of diamond particles to realize metallurgical bonding between diamond and the matrix. Meanwhile, the direct contact between Fe, Co, Ni and diamond particles can be avoided to prevent the formation of hard and brittle carbides at the interface [16,17]. Therefore, the ideal structure of metallurgical bonding between diamond particles and a metal matrix involves avoiding the formation of carbides.

In this paper, a novel HEA/diamond composite was studied by magnetron sputtering Ti/Ni coating and the spark plasma sintering (SPS) method. A Ti/Ni coating (inner Ti and outer Ni) of diamond particles was realized by magnetron sputtering. An HEA/coated diamond interface was obtained, composed of the solid solution formed by diffusion between the HEA matrix and Ni element in the outer layer of diamond particles. The carbide formed by the reaction of Ti element with diamond particles. On the one hand, this interface can effectively reduce the interface energy between diamond and the HEA matrix and improve the bonding strength. On the other hand, the cracks and micro-holes on the interface after SPS can be filled by generated carbides to improve the density of the composites and improve the strength, toughness and other mechanical properties of the composites.

## 2. Experimental

**Figure 1:**
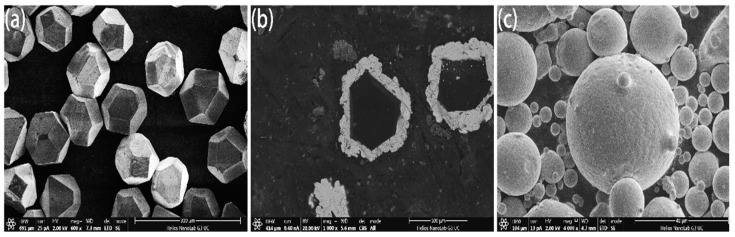
Microstructure of (**a**) uncoated and (**b**) coated diamond particles and (**c**) high-entropy alloy (HEA) powders.

Standard MBD4-type synthetic diamond single crystals of 140/170 mesh were boiled and rinsed in HNO_3_ and NaOH solutions for surface purification. Diamond was deposited in the magnetron sputtering coating equipment and high purity argon was introduced. The metals Ti and Ni with purity higher than 99.99% were used as targets. The diamond surface was coated by vacuum magnetron sputtering and maintained at room temperature. The vacuum of the reaction chamber was 10^−4^ Pa, the partial pressure of argon was 10^−1^ Pa, the ion deposition rate was 20 nm/min and the diamond was rolled by ultrasonic vibration in the diamond tray to ensure the uniformity of the coating. The thickness was controlled and was about 15 μm as shown in Figure 1. FeCoCrNiMo HEA powders prepared by gas atomization were applied as matrix materials as shown in Figure 1c, in which the average particle size was about 50 μm. Coated and uncoated diamond particles with a mass ratio of 4% were mixed with HEA powder and put into the mixer for 5 hours (mixer speed: 60 r/min). HEA/diamond composites were prepared by SPS (SPS parameters: 950 ℃/35 MPa, holding time: 480 s).

The density of samples was measured by the Archimedes drainage method. The transverse fracture strength of the samples (size: 12 × 2 × 30 mm^3^, span: 25 mm, loading rate: 2 mm/min) was determined by the Instron 3369 mechanical testing facility (Instron, Norwood, MA, USA) using the three-point method. The hardness of the alloy was determined using a Vickers hardness tester (200HVS-5, HuaYin, Zhengzhou) under a 200 g load for 15 s and was averaged from five measurements. The wearing behavior was measured by HRS-2M high-speed reciprocating line friction test equipment (Lanzhou Zhongke Kaihua Technology Development Co., Ltd., Lanzhou, China). The test parameters were a test time of 15 min, 50 N loading, 15 Hz frequency (900 times/min) and a 5-mm stroke. A scanning electron microscope (SEM, FEI, Quanta 250 FEG, Vlastimila Pecha, Czech Republic) equipped with an energy dispersive X-ray (EDX) analyzer was used to investigate the microstructure and chemical compositions of the sintered samples. Confocal Raman microscopy was performed on the interface of diamonds/HEA using an inVia Reflex by Renishaw with an Ar laser, using the green line (532 nm, 7.6 mW) with a resolution of 0.5 × 2 μm.

## 3. Results and Discussion

### 3.1. Interface of HEA/Diamond Composite

**Figure 2:**
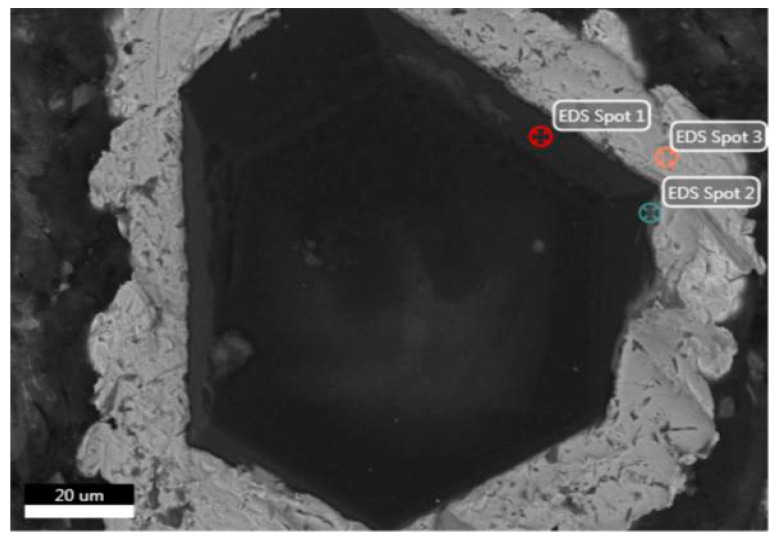
Microstructure of Ti/Ni-coated diamond particles.

In the SEM analysis of the coated diamond (Figure 2 and Table 1), spot 1 at the edge of the diamond shows all C atoms. Spot 2 is in the transition zone between the diamond and the coated layer. C, Ti and Ni elements can be found in the area near the diamond, of which the content of Ni is relatively small. Spot 3 is in the coated layer, whereas that of Ni element is much more in the outer layer. This is because Ti is a strong carbide-forming element with strong chemical activity and diffusion ability. During the SPS process, Ti reacts with diamond to form a stable, chemically bonded TiC layer. At the same time, because the matrix is a five-element HEA of FeCoCrNiMo, the higher amount of Ni element in the outer layer can form a solid solution interface with the HEA matrix.

**Figure 3:**
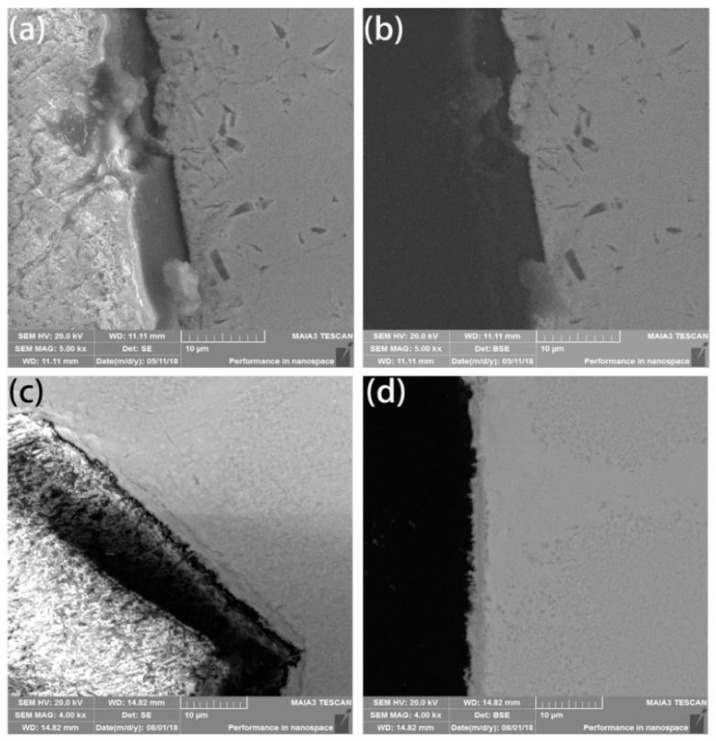
Scanning Electron Microscope (SEM) and Back Scattered Electron (BSE) images of (**a**,**b**) uncoated diamond particles and (**c**,**d**) coated diamond particles.

The density of coated diamond increases by about 1/3 due to its multi-layer. This is closer to the density of a high-entropy alloy matrix, which makes the mixture more uniform, reduces the number of voids and indirectly improves the density after sintering. The HEA matrix was found to form a metallurgical bonding with diamond and shows a thin layer with a width of approximately 3 μm at the interface between the diamond and the matrix. This indicates that the metallurgical bonding between the coated diamond particles and the matrix is relatively strong, as shown in Figure 3c,d, while the uncoated diamond resists mechanical bonding to a certain extent, as shown in Figure 3a,b.

**Figure 4:**
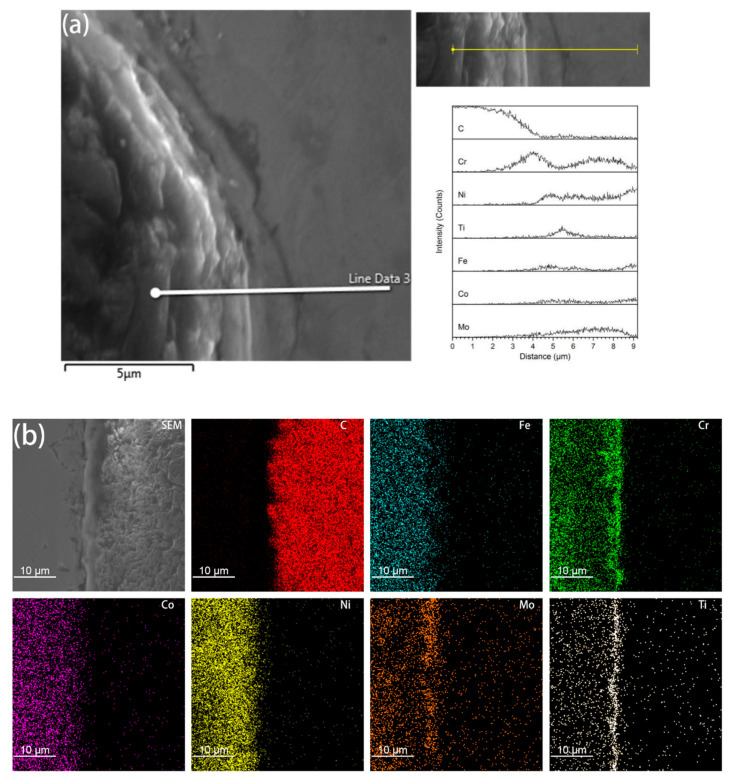
EDS liner scan (**a**) and element mapping (**b**) analysis on the interface of coated diamond/HEA composite.

It was found from the Energy Dispersive Spectrometer (EDS) analysis of the interface of coated diamond/HEA composites that, as shown in Figure 4, there is a high content of Cr and Fe elements between the diamond and the high-entropy alloy matrix, that is, the segregation of Cr, Ti, Ni and Mo elements occurs and the reaction with diamond produces a solid solution layer with a higher bonding strength than that achieved via pure mechanical bonding. It can also be seen from the image that although C atoms aggregate at the interface, they do not extend to the matrix. Although the functional metallic layer has a certain corrosion effect on diamond, it does not destroy the crystalline form of diamond to a large extent. On the contrary, the transition layer restricts the diffusion of carbon atoms to the matrix. It can be seen from Figure 3c,d that the transition layer is different from the non-functional diamond. The smooth bonding surface of diamond composites and the functional diamond composites show a fold structure. This structure increases the bonding area between diamond and the matrix in the transition layer, so that it can be better bonded with the matrix. Moreover, the EDS surface analysis of the interface between coated diamond and the matrix was carried out. As shown in Figure 4b, in accordance with the results of liner scanning, there is obvious segregation at the interface and obvious precipitation of Cr and Mo. This indicates that the interface layer may be a solid solution composed of Cr, Mo, Ti and Ni, indicating that the functionalized layer can form a metallurgical bond with the HEA matrix that will increase the interfacial bonding strength.

**Figure 5:**
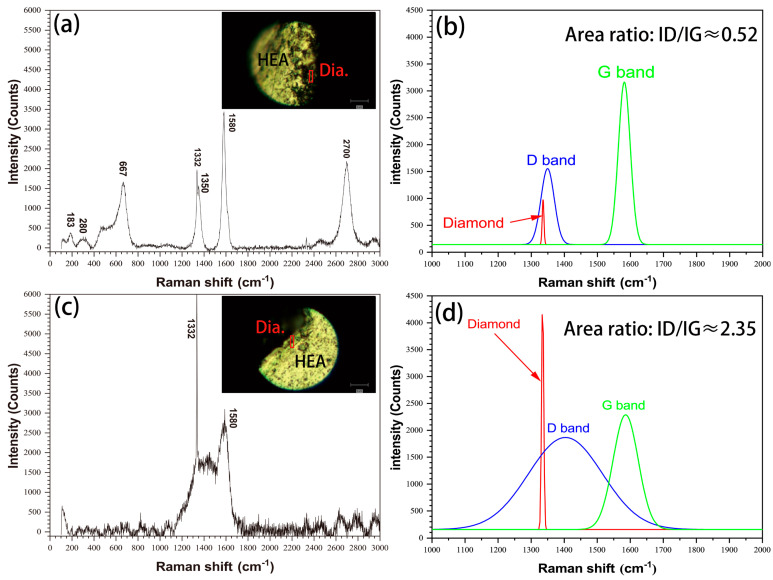
Raman spectra of HEA matrix/diamond interface in (**a**) uncoated diamond and (**c**) coated diamond, as well as the corresponding fitting curves (1000 cm^−1^ ~ 2000 cm^−1^) (**b**,**d**).

The interface status of HEA matrix/diamond were also characterized by Raman spectroscopy, as shown in Figure 5. As shown in Figure 5a, there are diamond peaks at 1332 cm^−1^, graphite peaks at 1350 cm^−1^ (D-band), 1580 cm^−1^ (G-band) and 2700 cm^−1^ (2D-band) and other bonds at the interface of the uncoated diamond specimens. The peak shape is sharp, the G-band strength is more than two times that of D-b (Figure 5b) and the area ratio of ID/IG (Intensity ratio of peak D to peak G) is about 0.52. The interface of the coated diamond specimens only exhibits a diamond peak at 1332 cm^−1^. For the peaks at 1350 cm^−1^ (D-band) and 1580 cm^−1^ (G-band) (Figure 5c), the peak shape is wide, the G-band strength and D-band strength are close (Figure 5d) and the area ratio of ID/IG is about 2.35, which is obviously higher than that of the uncoated diamond samples. The results show that the graphitization degree of diamond particles in uncoated diamond samples is higher [18] and the other bonds indicate that there are carbides or oxides at the interface [19]. In conclusion, coated diamond can effectively inhibit the graphitization structural transformation of diamond particles in the SPS process and improve the material properties. 

### 3.2. Microstructure of the HEA/Diamond Composite

**Figure 6:**
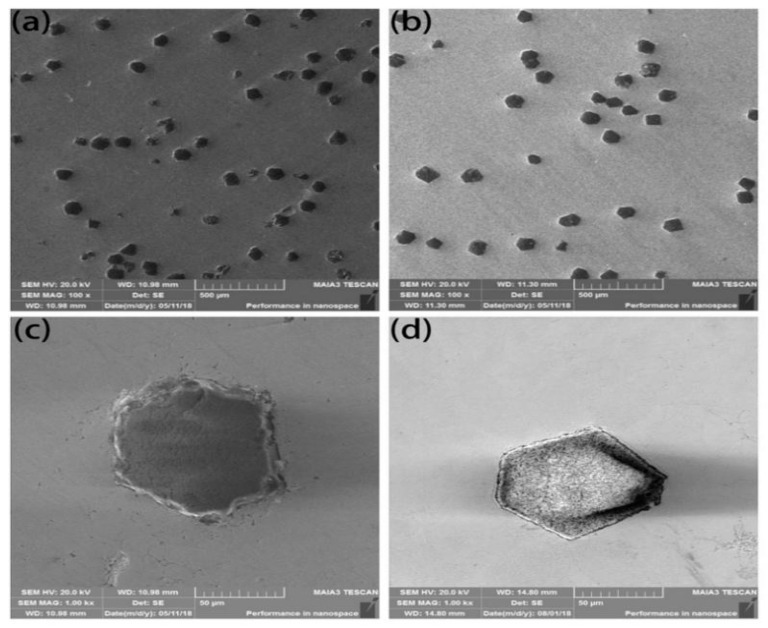
Microstructure of the composites fabricated by spark plasma sintering (SPS) with uncoated (**a**,**c**) and coated diamond (**b**,**d**).

The microstructure of the composites fabricated by SPS with coated and uncoated diamond is shown in Figure 6. Uncoated diamond particles are severely ablated. After sintering, the original diamond with a regular shape displays jagged ablation on its surface, which cannot maintain the original excellent crystal form of the diamond as shown in Figure 6a,c. The coated diamond also has a certain degree of ablation, as shown in Figure 6b,d. However, due to the passivation of the coated metal layer, which prevents the corrosion of the matrix to diamond and reduces the diffusion of C element to the matrix, a good crystal form of diamond is maintained. Meanwhile, the coating contains Ti element, while Ti element and C element form carbides and the second-phase carbides disperse in the matrix, thus improving the hardness of the FeCoCrNiMo high-entropy alloy system [20]. Therefore, the friction and wear properties of materials are also affected.

**Figure 7:**
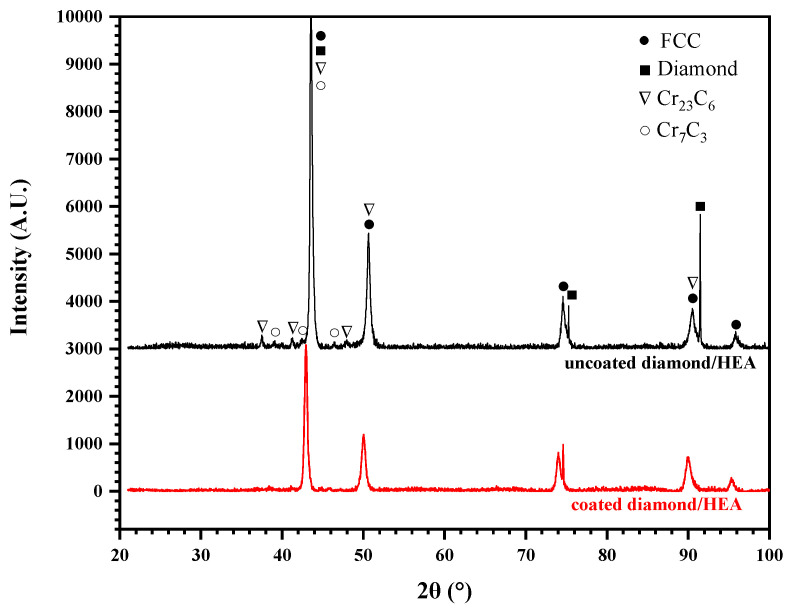
XRD pattern of the composites fabricated by SPS with coated and uncoated diamond.

According to the X-ray diffraction (XRD) analysis of the HEA/diamond composite, as shown in Figure 7, only two strong peaks of diamond were detected, because of the low content of diamond and the close distance between the Face-Centred Cubic (FCC)peak and the diamond peak of HEA. However, no carbide and C element were detected. This indicates that there was no large amount of ablation or graphitization of the diamond in coated diamond samples. Moreover, the HEA matrix had an FCC structure and there was no complex multiphase structure and no intermetallic compound found in coated samples in Figure 7. Therefore, the excellent properties of HEA matrix could be maintained. Yet, in uncoated diamond samples, the addition of diamond particles was found to form any complex carbide phase and there existed carbides of Cr according to the powder diffraction files (PDF), such as Cr23C6 (PDF No. 04-004-1672) and Cr7C3 (PDF No. 04-005-9649). These carbides were hard and brittle, which could deteriorate the mechanical properties of the composite. 

### 3.3. Mechanical Properties

From the density of uncoated diamond/HEA composites in Table 2, the density difference between uncoated diamond and HEA powder was found to be large, which not only causes inhomogeneity in the mixing process, but also causes a low density of green compacts. Under such circumstances, it is impossible to eliminate the inhomogeneous distribution of pressure in the sintered block caused by pores, which ultimately results in the insufficient density of composites. The surface coating of diamond can improve the density of diamond particles, make the mixture more uniform, reduce the number of pores and increase the sintering density. At the same time, the activation of diamond can promote the diffusion reaction between diamond and the matrix and produce good metallurgical bonding. According to the results of hardness tests (Table 2), it was found that the hardness of the samples of coated diamond composites is about 30 HV higher than the that of uncoated samples, because the graphitization of diamond particles (Figure 4c) in the uncoated diamond composites reduces the hardness of the matrix. 

The fracture strength of coated diamond composites is 745 MPa, which is far greater than that of uncoated diamond samples, as shown in Table 2. The reason for the low fracture strength of uncoated diamond composites is the low density caused by the micro-voids in the materials, the graphitization of diamond as a hard phase and the poor bonding between diamond and the HEA matrix. It does not produce good metallurgical bonding, but is only a metallurgical bonding and mechanical bonding interface.

**Figure 8:**
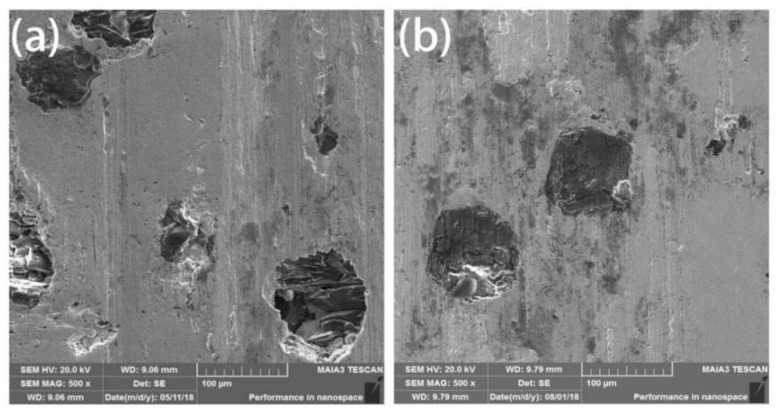
The wearing surface of the composites fabricated by SPS with uncoated (**a**) and coated (**b**) diamond.

Friction and wear performance is an important property of diamond tools. Comparing the SEM images after friction from the wear mechanism, as shown in Figure 8, there were ravines and trace debris on the surface of the both coated and uncoated diamond composites. It can be inferred that the wear mechanism is abrasive wear with partially adhesive wear. The scratches on the surface of uncoated diamond composites were obvious and diamond shedding occurred. Through the analysis of the exfoliated parts in Figure 8a,b, it was found that although the diamond and the matrix have a certain degree of metallurgical bonding, due to the loose structure and inadequate bonding, the diamond will fall off from the matrix and fail. Compared with the coated diamond composite, the coated diamond can be worn without showing signs of falling off (Figure 8c,d). This shows that the Ti/Ni coating can enhance the control ability of the HEA matrix over diamond particles, improve the bonding strength and thus enhance the service life of diamond composites.

**Figure 9:**
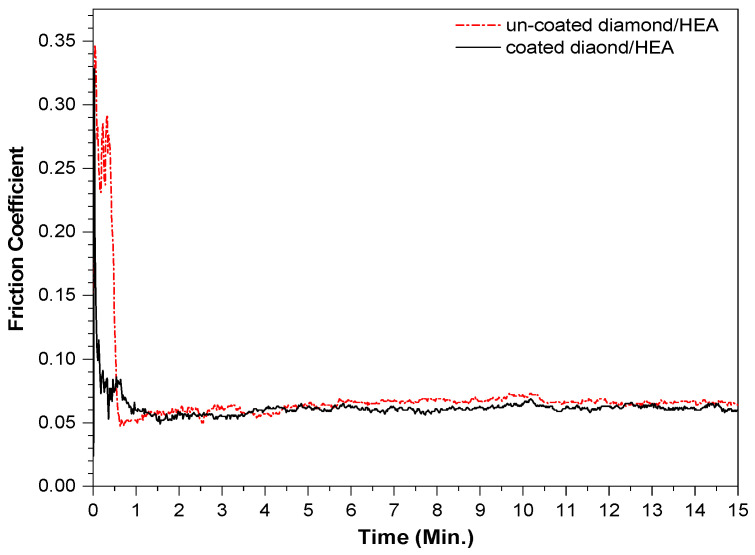
The friction coefficient curve of the composites fabricated by SPS with uncoated and coated diamond.

The friction coefficients of the two kinds of samples fabricated by SPS ranged from 0.04 to 0.06 and maintained a very stable trend, as shown in Figure 9. After 15 min of friction, the wear of the uncoated diamond samples was 0.0144 g, while the wear of the coated diamond samples was only 0.0127 g. This shows that the friction and wear properties of the two samples are both excellent, which also confirms that the hardness of the two samples is not very different. The reason for the smaller friction coefficient is that the diamond in the sample is relatively complete and bare. The agate ball only rubs on the diamond surface, while the strong control of the matrix on diamond keeps the friction coefficient in a stable linear trend. That is, in the actual friction process, diamond plays a major role in friction, while the HEA matrix holds on the diamond. The basic type of wear is the combination of abrasive wear and adhesive wear. In general, the friction failure of diamond tools is the shedding of diamond or the wear of matrix; however, the surface coating of diamond can effectively avoid the shedding of diamond particles.

## 4. Conclusions

Diamond single crystal particles with Ti/Ni coatings were obtained by magnetron sputtering. The diamond gained more than 30% weight and the coating thickness was 10–20 μm.

The interfacial diffusion and solid solution formation in coated diamond composites led to the increase of interfacial bonding strength. The interface bonding strength and mechanical properties of the composites without diamond coating decreased, due to the occurrence of graphitization at the interface and the formation of carbides.

Compared with the composite with uncoated diamond, the density, hardness, transverse fracture strength and interfacial bonding properties of the composite with coated diamond were significantly improved.

The wear resistance of the coated composites is obviously higher than that of the uncoated composites, because the coating on the diamond particles maintains a relatively complete crystal form in the SPS process and has a higher bonding strength with the HEA matrix. This improves the holding force of the matrix and gives full play to the excellent properties of the HEA.

## Figures and Tables

**Table 1:** Chemical composition of different spots in Figure 2.

Composition (wt.%)	C	Ti	Ni	P	O
Spot 1	100	/	/	/	/
Spot 2	59.29	30.64	6.57	3.08	0.42
Spot 3	3.96	0.76	91.09	3.72	0.47

**Table 2:** Density, hardness and transverse fracture strength of the composites fabricated by SPS with coated and uncoated diamond.

Composite Samples	Uncoated Diamond	Coated Diamond
Density(g/cm^3^)	7.072 ± 0.053	7.240 ± 0.046
HV_0.2_	342.2 ± 38.5	370.4 ± 34.8
Transverse fracture strength (MPa)	~550	~750
